# Investigating the causal roles of cholesterol and triglyceride subfractions across lipoproteins in irritable bowel syndrome: a Mendelian randomization study

**DOI:** 10.1016/j.metop.2025.100434

**Published:** 2025-12-18

**Authors:** Fang Peng, Kemin Xu, Qinwen Ba, Hao Bi, Yanjun Lu

**Affiliations:** a Department of Laboratory Medicine, Tongji Hospital, Tongji Medical College, Huazhong University of Science and Technology, Wuhan, China; b School of Laboratory Medicine, Hubei University of Chinese Medicine, 16 Huangjia Lake West Road, Wuhan, 430065, China; c Department of Laboratory Medicine, Maternal and Child Health Hospital of Hubei Province, Tongji Medical College, Huazhong University of Science and Technology, Wuhan, China

**Keywords:** Irritable bowel syndrome, Mendelian randomization, Cholesterol, Triglyceride, Causality

## Abstract

**Background:**

Dietary composition and nutrient intake are increasingly recognised as key modulators of symptom onset and severity in a substantial subset of patients with irritable bowel syndrome (IBS). Among dietary lipids, cholesterol and triglycerides have emerged as potential contributors. Nevertheless, the causal relationship remains poorly understood, and evidence in this area remains limited. To address this gap, we conducted a Mendelian randomization (MR) study to dissect the putative causal effects of cholesterol and triglycerides on IBS risk in individuals of European ancestry.

**Methods:**

Instrumental variables were constructed for 17 lipid traits—total cholesterol, free cholesterol, triglycerides, and 13 size-specific High-density lipoprotein(HDL), Low-density lipoprotein(LDL), and Very low-density lipoprotein (VLDL) sub-fractions—using summary-level data from the largest available genome-wide association studies (GWAS). Lipid exposures were derived from a meta-analysis of 115,082 European-ancestry individuals. IBS outcomes were ascertained in an independent cohort of 486,601 participants of European descent. Causal estimates were obtained with five complementary MR methods: inverse-variance weighted (IVW), weighted median, MR-Egger, simple mode, and weighted mode. Robustness was further interrogated with leave-one-out permutation, MR-Egger intercept evaluation of directional pleiotropy, and Cochran's Q assessment of heterogeneity.

**Results:**

The MR analysis indicated that cholesterol or free cholesterol content of chylomicrons and extremely large VLDL particles were causally associated with an increased risk of developing IBS (β = 0.0679, odds ratios (OR) = 1.070, 95 % CI 1.015–1.128, P = 0.0118; and β = 0.0650, OR = 1.0672, 95 % CI 1.0179–1.1189, P = 0.0007, respectively). Conversely, equivalent increments in cholesterol or free cholesterol carried by very large HDL or very small VLDL conferred protection (β = −0.0626, OR = 0.9392, 95 % CI 0.8992–0.9810, P = 0.0047; and β = -0.0604, OR = 0.9412, 95 % CI 0.9023–0.9819, P = 0.005). Total circulating triglycerides also displayed a positive causal effect on IBS susceptibility (β = 0.0798, OR = 1.083, 95 % CI 1.0268–1.1423, P = 0.0033), with concordant directional estimates observed across the majority of lipoprotein sub-fractions.

**Conclusion:**

Whereas triglycerides conveyed a uniformly adverse effect on IBS risk regardless of lipoprotein carrier, cholesterol displayed a net protective signal across most particle classes. This dichotomy implies that lipid-centred interventions should discriminate between the two moieties: dietary or pharmacological strategies that selectively lower triglycerides while preserving—or even augmenting—cholesterol in specific lipoprotein fractions may offer a rational, mechanistically grounded approach to IBS prevention and management.

## 1 Introduction

Irritable bowel syndrome (IBS) is the most common functional gastrointestinal disorder, impairing quality of life and generating substantial direct and indirect health-care costs. Meta-analyses report a global prevalence of 7–21 %, and incidence continues to rise [1]. The disorder is defined by recurrent abdominal pain or discomfort accompanied by bloating, visible distension and altered bowel habits—diarrhoea, constipation or a mixed pattern—in the absence of detectable structural or biochemical abnormalities [2]. Although the mechanisms underlying IBS are still unclear, converging evidence highlights abnormal gut motility, visceral hypersensitivity [3], disturbed gut–brain communication [4], genetic predisposition, low-grade mucosal inflammation and alterations in the intestinal microbiota [5].

Up to 80 % of patients attribute symptom onset or exacerbation to specific foods. Systematic reviews indicate that obesity increases IBS risk: affected individuals have higher mean body-mass index than healthy controls [6,7]. Proposed mechanisms include malabsorption of fermentable carbohydrates and lipids, together with obesity-related sub-clinical inflammation that heightens colonic sensitivity [8]. Comparative metabolomic profiling has repeatedly revealed divergent plasma lipid signatures in IBS, with triglycerides, cholesterol, free fatty acids and related metabolites consistently elevated in patients versus controls [9,10]. Hypertriglyceridaemia may reflect low-grade mucosal inflammation, increased intestinal permeability and dysmotility that collectively impair fat absorption [11]. Consistent with this observation, lipid-lowering therapy can ameliorate IBS symptoms [12]. Nevertheless, cross-sectional and case-control studies cannot disentangle whether lipid disturbances predispose to, or result from, the disorder.

Mendelian randomization (MR) uses germline genetic variation as a proxy for lifetime exposure, providing unconfounded estimates of causal associations [13]. Although MR has clarified the contribution of Low-density lipoprotein (LDL) cholesterol, triglycerides and High-density lipoprotein (HDL) cholesterol to cardiometabolic disease, analogous investigations for IBS are scarce. We therefore conducted a comprehensive two-sample MR study to quantify the causal effects of total cholesterol, triglycerides and lipoprotein sub-fractions on IBS risk, with the goal of informing precision dietary or pharmacological prevention strategies.

## 2 Methods

### 2.1 Study overview

We conducted a two-sample MR investigation to estimate the causal effects of 17 lipids (total cholesterol, free cholesterol, cholesteryl esters, triglycerides and 13 size-resolved HDL, LDL and Very low-density lipoprotein (VLDL) sub-fractions on IBS. The design satisfies the three core MR assumptions: (i) genotype–exposure association, (ii) independence from confounders and (iii) exclusion-restriction (no pathway to the outcome except via the exposure). Analyses were restricted to individuals of European ancestry to minimise population stratification. Exposure estimates were derived from a discovery GWAS (n = 115,082) and replicated in an independent validation cohort, outcome data comprised 486,601 IBS cases (Table S1–S3).

### 2.2 Data sources

Summary-level GWAS statistics for all lipid traits were downloaded from the IEU OpenGWAS repository (accessed July 1, 2024). The discovery lipid GWAS included 11,590,339 Single-nucleotide polymorphisms (SNPs), the IBS summary statistics comprised 9,739,966 SNPs. All datasets were procured from the IEU OpenGWAS platform(https://gwas.mrcieu.ac.uk/).All contributing participants self-reported white European ancestry. Ethical approval and informed consent were obtained in each original study,the present analysis used only publicly available de-identified data.

### 2.3 Instrument selection

SNPs associated with each lipid at genome-wide significance (P < 5 × 10^−8^) were clumped (r^2^ < 0.001 within 10 Mb) using the 1000 Genomes European reference panel via the TwoSampleMR package (v 0.4.25). Palindromic SNPs with ambiguous strands were removed; proxies (r^2^ > 0.8) were retrieved when the lead SNP was unavailable. Instrument strength was quantified by R^2^ and F-statistics; only variants with F > 10 were retained, ensuring adequate power and minimising weak-instrument bias.

### 2.4 Primary and sensitivity analyses

Causal estimates were obtained with five complementary methods: inverse-variance weighted (IVW, primary estimator), MR-Egger, weighted median, simple mode and weighted mode. IVW provides the most precise estimate under the no-pleiotropy assumption; MR-Egger regression was used to test for and correct directional pleiotropy (intercept P < 0.05 considered indicative). The weighted median estimator remains consistent even if up to 50 % of the weight derives from invalid instruments. Cochran's Q statistic was calculated to quantify heterogeneity,a P-value >0.05 implies homogeneity. Leave-one-out analysis was performed to verify that no single SNP drove the findings. Results are reported as odds ratios (OR) per 1-standard-deviation increase in genetically predicted lipid levels with 95 % confidence intervals (CI).

### 2.5 Statistical analysis

The statistical analyses were meticulously conducted utilizing the TwoSampleMR package (v0.4.25) within the R environment (v3.6.2).

## 3 Results

### 3.1 Instrument strength

Following clumping and harmonisation, all instrumental variants achieved genome-wide significance (P < 5 × 10^−8^) and F-statistics >10 (Table S4), indicating robust instruments with minimal weak-instrument bias.

### 3.2 Influence of total lipids on IBS

Genetically predicted total cholesterol, esterified cholesterol and free cholesterol showed no causal effect on IBS. (β = −0.0385, OR = 0.9622, 95 %CI = 0.9188–1.0076, P = 0.1016; β = −0.0456, OR = 0.9553, 95 %CI = 0.9104–1.0025, P = 0.0631; β = −0.031, OR = 0.9693, 95 %CI = 0.9219–1.0192, P = 0.2247) (Table 1). In contrast, each 1-SD increase in total triglycerides conferred a 8.3 % higher risk (IVW OR = 1.083, 95 % CI 1.027–1.142, P = 0.003), corroborated by the weighted-median estimator (β = 0.0643, OR = 1.0665, 95 %CI = 1.0063–1.1302, P = 0.0296). MR-Egger intercept provided no evidence of directional pleiotropy for total triglycerides and IBS (intercept = 0.0017, P = 0.4965), although Cochran's Q indicated significant heterogeneity (Q = 145.123695, P = 7.15 × 10^−8^) (Table 1).

**Table 1:** Associations of genetically determined cholesterol levels, esterified cholesterol, and free total cholesterol, and triglyceride levels with Irritable bowel syndrome.

	Mendelian Randomization Method	No. of SNPs used	β	OR	95 % CI	P value	Pleiotropy test		Heterogeneity test	
							MR-Egger intercept	P value	MR Egger (Q)	P value
Total cholesterol levels	IVW	60	−0.0385	0.9622	0.9188,1.0076	0.1016	0.0006	0.7848	85.3495	0.0112
	MR Egger		−0.0464	0.9546	0.8869,1.0273	0.22				
	Weighted median		−0.0587	0.9429	0.8846,1.005	0.0709				
	Weighted mode		−0.0538	0.9475	0.8959,1.0021	0.0642				
	Simple mode		−0.0499	0.9513	0.8392,1.0782	0.438				
Total esterified cholesterol levels	IVW	61	−0.0456	0.9553	0.9104,1.0025	0.0631	−0.001	0.6519	94.5801	0.0022
	MR Egger		−0.032	0.9684	0.8972,1.0454	0.415				
	Weighted median		−0.0586	0.943	0.8853,1.0045	0.0687				
	Weighted mode		−0.0547	0.9467	0.8916,1.0051	0.0781				
	Simple mode		−0.1153	0.891	0.7813,1.0162	0.0906				
Total free cholesterol levels	IVW	62	−0.031	0.9693	0.9219,1.0192	0.2247	0.0013	0.5618	105.3697	0.0002
	MR Egger		−0.0492	0.9519	0.8795,1.0303	0.2274				
	Weighted median		−0.0613	0.9405	0.8804,1.0046	0.0685				
	Weighted mode		−0.0577	0.9439	0.8868,1.0046	0.0746				
	Simple mode		−0.0775	0.9253	0.8131,1.053	0.2441				
Total triglycerides levels	IVW	68	0.0798	1.083	1.0268,1.1423	0.0033	0.0017	0.4965	145.1236	7.15E-8
	MR Egger		0.0565	1.0581	0.9713,1.1526	0.2002				
	Weighted median		0.0643	1.0665	1.0063,1.1302	0.0296				
	Weighted mode		0.0517	1.053	0.9933,1.1163	0.087				
	Simple mode		0.0623	1.0643	0.9541,1.1872	0.2675				

### 3.3 Lipids within HDL, LDL and VLDL classes on IBS

Cholesterol or free cholesterol content of HDL, LDL or VLDL particles was not causally related to IBS (P > 0.05 for all estimators). Triglyceride cargo of VLDL, however, replicated the adverse signal observed for total triglycerides (IVW OR = 1.0843, 95 % CI 1.0297–1.1418, P = 0.0021), with no indication of pleiotropy (Egger intercept P = 0.38) but significant heterogeneity (Q P < 0.001) (Table 2).

**Table 2:** Associations of genetically determined total or free cholesterol levels, triglycerides in HDL, LDL and VLDL with Irritable bowel syndrome.

	Mendelian Randomization Method	No. of SNPs used	β	OR	95 % CI	P value	Pleiotropy test		Heterogeneity test	
							MR-Egger intercept	P value	MR Egger (Q)	P value
Cholesterol levels in HDL	IVW	86	−0.0359	0.9647	0.9204,1.0111	0.1339	−0.00006	0.9777	159.4851	1.31E-06
	MR Egger		−0.035	0.9655	0.893,1.044	0.3818				
	Weighted median		−0.0132	0.9867	0.9309,1.0459	0.6546				
	Weighted mode		−0.0326	0.9678	0.918,1.0203	0.2289				
	Simple mode		−0.0224	0.9777	0.8719,1.0963	0.7011				
Cholesterol levels in LDL	IVW	46	−0.028	0.9723	0.9299,1.0166	0.2168	0.0023	0.3047	65.4735	0.0194
	MR Egger		−0.0511	0.9501	0.8928,1.0111	0.1146				
	Weighted median		−0.0472	0.9538	0.903,1.0075	0.0911				
	Weighted mode		−0.0471	0.9539	0.9077,1.0024	0.069				
	Simple mode		−0.0631	0.9388	0.8284,1.0639	0.328				
Cholesterol levels in VLDL	IVW	67	−0.0269	0.9734	0.9193,1.0307	0.3565	−0.0017	0.5397	142.6625	9.52E-08
	MR Egger		0.0007	1.0007	0.9008,1.1117	0.9883				
	Weighted median		−0.0189	0.9811	0.9193,1.0471	0.5674				
	Weighted mode		−0.0438	0.957	0.8868,1.0328	0.263				
	Simple mode		−0.0491	0.952	0.8396,1.0794	0.4456				
Free cholesterol levels in HDL	IVW	80	−0.0234	0.9767	0.933,1.0226	0.3154	−0.0014	0.5155	141.6534	1.4119E-05
	MR Egger		−0.0033	0.9966	0.9237,1.0752	0.9311				
	Weighted median		−0.0119	0.9881	0.9378,1.041	0.6529				
	Weighted mode		−0.0166	0.9835	0.9403,1.0286	0.4699				
	Simple mode		−0.0213	0.9788	0.8725,1.0982	0.717				
Free cholesterol levels in LDL	IVW	48	−0.04	0.9606	0.9196,1.0035	0.0717	0.0002	0.9284	77.1396	0.0027
	MR Egger		−0.0419	0.9589	0.9034,1.0178	0.1745				
	Weighted median		−0.042	0.9587	0.9164,1.003	0.0676				
	Weighted mode		−0.0387	0.9619	0.9193,1.0066	0.1004				
	Simple mode		−0.0105	0.9895	0.8822,1.1099	0.858				
Free cholesterol levels in VLDL	IVW	61	0.0066	1.0066	0.9489,1.0679	0.8251	−0.0018	0.5423	135.4633	6.00334E-08
	MR Egger		0.0351	1.0357	0.929,1.1545	0.5289				
	Weighted median		0.0081	1.0081	0.9453,1.0752	0.8043				
	Weighted mode		0.0105	1.0106	0.9409,1.0854	0.7728				
	Simple mode		0.0105	1.0106	0.8932,1.1434	0.8673				
Triglyceride levels in HDL	IVW	58	0.0455	1.0466	0.9984,1.0971	0.0579	0.0038	0.1437	118.0013	2.55825E-06
	MR Egger		0.0049	1.005	0.9360,1.0790	0.891				
	Weighted median		−0.0003	0.9996	0.9474,1.0548	0.9909				
	Weighted mode		0.0086	1.0087	0.9642,1.0552	0.7075				
	Simple mode		0.0167	1.0168	0.9190,1.1250	0.7472				
Triglyceride levels in LDL	IVW	72	0.0313	1.0318	0.9742,1.0928	0.2848	−0.0007	0.7932	200.0535	1.86158E-14
	MR Egger		0.0410	1.0419	0.9497,1.1431	0.3880				
	Weighted median		−0.0087	0.9912	0.9369,1.0487	0.7610				
	Weighted mode		−0.0038	0.9961	0.9459,1.0491	0.8857				
	Simple mode		0.0569	1.0586	0.9449,1.1860	0.3290				
Triglyceride levels in VLDL	IVW	68	0.0809	1.0843	1.0297,1.1418	0.0021	0.0021	0.3768	135.5063	1.02935E-06
	MR Egger		0.0527	1.0541	0.9722,1.1429	0.2058				
	Weighted median		0.0709	1.0735	1.0123,1.1385	0.0178				
	Weighted mode		0.0562	1.0578	0.9977,1.1216	0.064				
	Simple mode		0.0882	1.0922	0.9805,1.2167	0.1135				

### 3.4 Influence of the cholesterol and triglyceride in different particle sizes of HDL, LDL, VLDL on IBS

Among 13 size-resolved fractions, cholesterol or free cholesterol in chylomicrons and extremely large VLDL increased IBS risk by ∼7 %(β = 0.0679, OR = 1.0703, 95 %CI = 1.0151–1.1284, P = 0.0118; β = 0.065, OR = 1.0672, 95 %CI = 1.0179–1.1189, P = 0.0070). Conversely, cholesterol in very large HDL, medium VLDL and very small VLDL conferred protection (β = −0.0626, OR = 0.9392, 95 %CI = 0.8992–0.9810, P = 0.0047; β = −0.0509, OR = 0.9503, 95 %CI = 0.9075–0.9951, P = 0.0302; β = −0.0604, OR = 0.9412, 95 %CI = 0.9023–0.9819, P = 0.0050),as did free cholesterol in very large HDL and very small VLDL (OR = 0.9259, P = 0.0007; OR = 0.9472, P = 0.0154) (Table 3, Table 4). Signals for cholesterol in small HDL and free cholesterol in small LDL were detected in the discovery cohort but were null in the validation cohort (Table S5 and S6).

**Table 3:** Associations of genetically determined cholesterol levels in different particle sizes of HDL, LDL and VLDL with Irritable bowel syndrome.

	Mendelian Randomization Method	No. of SNPs used	β	OR	95 % CI	P value	Pleiotropy test		Heterogeneity test	
							MR-Egger intercept	P value	MR Egger (Q)	P value
Cholesterol levels in chylomicrons and extremely large VLDL	IVW	65	0.0679	1.0703	1.0151,1.1284	0.0118	0.001	0.6972	146.7685	1.23E-08
	MR Egger		0.0553	1.0568	0.9728,1.1481	0.1953				
	Weighted median		0.0506	1.0519	0.9951,1.112	0.0734				
	Weighted mode		0.0484	1.0496	0.9959,1.1062	0.0752				
	Simple mode		0.0104	1.0105	0.9124,1.1191	0.8414				
Cholesterol levels in very large HDL	IVW	91	−0.0626	0.9392	0.8992,0.981	0.0047	−0.0008	0.6807	176.0231	1.12E-07
	MR Egger		−0.0523	0.949	0.8886,1.0134	0.1218				
	Weighted median		−0.04	0.9607	0.9107,1.0134	0.1415				
	Weighted mode		−0.0555	0.9459	0.9088,0.9846	0.0078				
	Simple mode		−0.0949	0.9094	0.8187,1.0101	0.0799				
Cholesterol levels in very large VLDL	IVW	57	0.0381	1.0388	0.9777,1.1038	0.2177	−0.0019	0.5275	141.7137	1.38E-09
	MR Egger		0.0647	1.0668	0.9632,1.1815	0.2195				
	Weighted median		0.0221	1.0224	0.9597,1.0891	0.4915				
	Weighted mode		0.0299	1.0304	0.9727,1.0916	0.3123				
	Simple mode		0.0195	1.0197	0.9177,1.1331	0.7172				
Cholesterol levels in large HDL	IVW	105	−0.0389	0.9617	0.9209,1.0044	0.0786	0.0008	0.6336	199.3181	4.04E-08
	MR Egger		−0.0516	0.9496	0.8873,1.0163	0.1388				
	Weighted median		−0.0254	0.9748	0.9274,1.0247	0.3167				
	Weighted mode		−0.0438	0.957	0.9135,1.0027	0.0678				
	Simple mode		−0.0734	0.9291	0.8353,1.0336	0.1794				
Cholesterol levels in large LDL	IVW	49	−0.0291	0.9712	0.9266,1.018	0.2246	0.0013	0.5712	82.0422	0.0011
	MR Egger		−0.0425	0.9583	0.8971,1.0237	0.2131				
	Weighted median		−0.0463	0.9546	0.9054,1.0066	0.0861				
	Weighted mode		−0.0436	0.9572	0.9102,1.0066	0.0952				
	Simple mode		−0.0617	0.9401	0.8368,1.0561	0.3038				
Cholesterol levels in large VLDL	IVW	57	0.0426	1.0435	0.9774,1.1142	0.2014	−0.0013	0.6879	162.4942	1.49E-12
	MR Egger		0.061	1.0629	0.9513,1.1875	0.2853				
	Weighted median		0.0129	1.013	0.9497,1.0804	0.6942				
	Weighted mode		0.0217	1.0219	0.9577,1.0905	0.5145				
	Simple mode		0.0079	1.0079	0.902,1.1263	0.8886				
Cholesterol levels in medium HDL	IVW	81	−0.0064	0.9935	0.9449,1.0446	0.7998	−0.0015	0.5121	149.0739	3.21E-06
	MR Egger		0.0171	1.0173	0.933,1.1092	0.6985				
	Weighted median		−0.005	0.9949	0.9339,1.0599	0.8762				
	Weighted mode		−0.0139	0.9861	0.924,1.0523	0.6744				
	Simple mode		0.0001	1.0001	0.8918,1.1214	0.9985				
Cholesterol levels in medium LDL	IVW	47	−0.0208	0.9793	0.9251,1.0366	0.4712	0.0018	0.5084	101.2406	3.25E-06
	MR Egger		−0.0421	0.9587	0.8809,1.0434	0.3346				
	Weighted median		−0.0514	0.9498	0.896,1.0068	0.0835				
	Weighted mode		−0.0484	0.9526	0.9033,1.0047	0.0805				
	Simple mode		−0.0243	0.9759	0.8606,1.1066	0.7062				
Cholesterol levels in medium VLDL	IVW	50	−0.0509	0.9503	0.9075,0.9951	0.0302	−0.0003	0.8808	69.859	0.0213
	MR Egger		−0.0468	0.9542	0.8889,1.0243	0.2014				
	Weighted median		−0.0587	0.9429	0.8864,1.0029	0.0621				
	Weighted mode		−0.0487	0.9523	0.8979,1.0101	0.1108				
	Simple mode		−0.0075	0.9924	0.8951,1.1003	0.8861				
Cholesterol levels in small HDL	IVW	44	0.0822	1.0856	1.0033,1.1748	0.0411	−0.00219	0.6215	118.923	2.8E-09
	MR Egger		0.1151	1.122	0.9635,1.3066	0.1458				
	Weighted median		0.1188	1.1261	1.0427,1.2162	0.0024				
	Weighted mode		0.133	1.1423	1.0554,1.2363	0.0019				
	Simple mode		−0.063	0.9389	0.773,1.1404	0.5287				
Cholesterol levels in small LDL	IVW	52	−0.0257	0.9745	0.925,1.0268	0.3337	0.0025	0.3118	106.861	5.22E-06
	MR Egger		−0.0527	0.9486	0.8814,1.0209	0.1659				
	Weighted median		−0.0475	0.9535	0.9037,1.0061	0.0827				
	Weighted mode		−0.0481	0.9529	0.9089,0.999	0.0509				
	Simple mode		−0.0232	0.977	0.87,1.0971	0.6960				
Cholesterol levels in small VLDL	IVW	68	−0.0356	0.9649	0.9156,1.0168	0.1819	−0.0012	0.6219	131.6731	2.86E-06
	MR Egger		−0.0166	0.9834	0.8971,1.0781	0.7234				
	Weighted median		−0.0216	0.9786	0.9215,1.0392	0.4809				
	Weighted mode		−0.0469	0.9541	0.8963,1.0157	0.1460				
	Simple mode		−0.0079	0.992	0.8863,1.1104	0.8904				
Cholesterol levels in very small VLDL	IVW	62	−0.0604	0.9412	0.9023,0.9819	0.0050	−0.0028	0.1729	88.2473	0.0102
	MR Egger		−0.0259	0.9744	0.9134,1.0394	0.435				
	Weighted median		−0.0289	0.9714	0.9205,1.0251	0.2914				
	Weighted mode		−0.0327	0.9677	0.9218,1.016	0.192				
	Simple mode		−0.0012	0.9987	0.8979,1.1107	0.9810

**Table 4:** Associations of genetically determined free cholesterol levels in different particle sizes of HDL, LDL and VLDL with Irritable bowel syndrome.

	Mendelian Randomization Method	No. of SNPs used	β	OR	95 % CI	P value	Pleiotropy test		Heterogeneity test	
							MR-Egger intercept	P value	MR Egger (Q)	P value
Free cholesterol levels in chylomicrons and extremely large VLDL	IVW	64	0.065	1.0672	1.0179,1.1189	0.0070	0.0005	0.8351	115.6974	4.21E-05
	MR Egger		0.0591	1.0609	0.9859,1.1416	0.1188				
	Weighted median		0.0729	1.0757	1.0196,1.1348	0.0075				
	Weighted mode		0.0529	1.0543	0.9974,1.1146	0.0663				
	Simple mode		0.0164	1.0165	0.9134,1.1313	0.7643				
Free cholesterol levels in very large HDL	IVW	77	−0.0769	0.9259	0.8853,0.9683	0.0007	−0.0014	0.4933	147.3818	1.23E-06
	MR Egger		−0.0591	0.9425	0.8807,1.0086	0.091				
	Weighted median		−0.0591	0.9303	0.8846,0.9783	0.0049				
	Weighted mode		−0.062	0.9398	0.899,0.9824	0.0076				
	Simple mode		−0.0949	0.9094	0.8131,1.0171	0.1005				
Free cholesterol levels in very large VLDL	IVW	60	0.0487	1.0499	0.9941,1.1088	0.0802	0.0027	0.8333	126.0482	5.99E-07
	MR Egger		0.0561	1.0578	0.9681,1.1557	0.2187				
	Weighted median		0.036	1.0366	0.9783,1.0985	0.2228				
	Weighted mode		0.0312	1.0317	0.9735,1.0934	0.2961				
	Simple mode		−0.0367	0.9639	0.8658,1.0731	0.5048				
Free cholesterol levels in large HDL	IVW	91	−0.0439	0.957	0.9136,1.0024	0.0633	0.0005	0.8001	191.2303	1.92E-09
	MR Egger		−0.0509	0.9503	0.8847,1.0207	0.1658				
	Weighted median		−0.0307	0.9696	0.921,1.0208	0.2404				
	Weighted mode		−0.042	0.9588	0.9196,0.9996	0.0513				
	Simple mode		−0.0672	0.9349	0.845,1.0344	0.1958				
Free cholesterol levels in large LDL	IVW	59	−0.0439	0.957	0.9151,1.0007	0.0541	−0.0009	0.6685	99.4248	0.0004
	MR Egger		−0.0346	0.9659	0.9079,1.0276	0.2776				
	Weighted median		−0.0438	0.957	0.9094,1.0072	0.092				
	Weighted mode		−0.0437	0.9572	0.9145,1.0018	0.0652				
	Simple mode		−0.0566	0.9449	0.8263,1.0805	0.4112				
Free cholesterol levels in large VLDL	IVW	59	0.0279	1.0516	0.9955,1.1108	0.0718	−0.0008	0.7771	124.409	6.32E-07
	MR Egger		0.0469	1.0628	0.9695,1.1652	0.1988				
	Weighted median		0.0304	1.0161	0.9573,1.0785	0.5991				
	Weighted mode		0.0323	1.0299	0.9666,1.0972	0.3658				
	Simple mode		0.0595	0.9863	0.8776,1.1085	0.818				
Free cholesterol levels in medium HDL	IVW	78	−0.0049	0.995	0.949,1.0432	0.8377	−0.0015	0.4852	132.5018	6.48E-05
	MR Egger		0.0185	1.0186	0.9394,1.1045	0.6552				
	Weighted median		−0.0088	0.9912	0.935,1.0508	0.7675				
	Weighted mode		−0.0202	0.9799	0.9317,1.0306	0.434				
	Simple mode		−0.0154	0.9846	0.887,1.0929	0.7719				
Free cholesterol levels in medium LDL	IVW	49	−0.0325	0.9679	0.9267,1.011	0.143	0.0011	0.584	76.9541	3.70E-03
	MR Egger		−0.0435	0.9573	0.9026,1.0153	0.1531				
	Weighted median		−0.0423	0.9585	0.9135,1.0057	0.0841				
	Weighted mode		−0.0414	0.9594	0.9148,1.0061	0.0941				
	Simple mode		−0.0212	0.9789	0.8698,1.1016	0.7253				
Free cholesterol levels in medium VLDL	IVW	64	−0.033	0.9674	0.9187,1.0186	0.2089	0.0008	0.7308	112.3657	9.55E-05
	MR Egger		−0.0452	0.9557	0.8767,1.0419	0.3082				
	Weighted median		−0.0508	0.9503	0.8943,1.0099	0.1006				
	Weighted mode		−0.0443	0.9566	0.8973,1.0198	0.1795				
	Simple mode		−0.0443	0.9566	0.8517,1.0744	0.4574				
Free cholesterol levels in small HDL	IVW	49	0.043	1.0439	0.9705,1.1228	0.2472	−0.0053	0.1773	111.0281	4.25E-07
	MR Egger		0.1312	1.1402	0.9858,1.3187	0.0835				
	Weighted median		0.0677	1.0701	0.9832,1.1647	0.1168				
	Weighted mode		0.1182	1.1254	1.001,1.2653	0.0537				
	Simple mode		0.0104	1.0104	0.832,1.2271	0.9167				
Free cholesterol levels in small LDL	IVW	51	−0.0369	0.9637	0.9304,0.9982	0.0396	−0.0004	0.789	64.6133	6.66E-02
	MR Egger		−0.0329	0.9676	0.924,1.0131	0.1672				
	Weighted median		−0.0364	0.9642	0.9248,1.0053	0.087				
	Weighted mode		−0.0339	0.9666	0.929,1.0056	0.0991				
	Simple mode		−0.0023	0.9976	0.8972,1.1092	0.965				
Free cholesterol levels in small VLDL	IVW	59	−0.0394	0.9612	0.9127,1.0123	0.135	−0.0004	0.8554	106.6864	7.39E-05
	MR Egger		−0.033	0.9674	0.8876,1.0543	0.454				
	Weighted median		−0.0548	0.9465	0.8905,1.0061	0.0777				
	Weighted mode		−0.0442	0.9566	0.9044,1.0118	0.1271				
	Simple mode		−0.0271	0.9732	0.8718,1.0863	0.6303				
Free cholesterol levels in very small VLDL	IVW	62	−0.0542	0.9472	0.9065,0.9897	0.0154	−0.0014	0.5264	96.9097	0.0017
	MR Egger		−0.0359	0.9647	0.8981,1.0362	0.3289				
	Weighted median		−0.0246	0.9756	0.9242,1.03	0.3732				
	Weighted mode		−0.0273	0.9729	0.9238,1.0247	0.3046				
	Simple mode		−0.032	0.9684	0.8755,1.0711	0.5348				

Triglycerides within almost every VLDL sub-fraction—and in small HDL, small LDL—were causally associated with higher IBS risk, respectively (OR range 1.067–1.096, all P < 0.05) (Table 5). Leave-one-out plots confirmed that no single SNP drove these associations.

**Table 5:** Associations of genetically determined triglyceride levels in different particle sizes of HDL, LDL and VLDL with Irritable bowel syndrome.

	Mendelian Randomization Method	No. of SNPs used	β	OR	95 % CI	P value	Pleiotropy test		Heterogeneity test	
							MR-Egger intercept	P value	MR Egger (Q)	P value
Triglyceride levels in chylomicrons and extremely large VLDL	IVW	60	0.08	1.0833	1.0325,1.1367	0.001	0.0028	0.2297	97.9418	8.00E-04
	MR Egger		0.0465	1.0476	0.9745,1.1261	0.2123				
	Weighted median		0.0659	1.0681	1.0064,1.1335	0.0298				
	Weighted mode		0.0567	1.0583	0.9975,1.1229	0.0652				
	Simple mode		0.0191	1.0193	0.9096,1.1422	0.7429				
Triglyceride levels in very large HDL	IVW	58	0.0066	1.0067	0.9600,1.0556	0.7821	−0.00004	0.9881	128.6338	1.21E-07
	MR Egger		0.0071	1.0071	0.9359,1.0838	0.8497				
	Weighted median		−0.0142	0.9858	0.9418,1.0318	0.5400				
	Weighted mode		−0.0008	0.9991	0.9566,1.0436	0.9705				
	Simple mode		0.0284	1.0288	0.9235,1.1461	0.6076				
Triglyceride levels in very large VLDL	IVW	76	0.0913	1.0957	1.0377,1.1568	0.0009	0.0032	0.2054	165.4466	6.02E-09
	MR Egger		0.0476	1.0487	0.9621,1.1432	0.2823				
	Weighted median		0.0752	1.0781	1.0165,1.1435	0.0122				
	Weighted mode		0.0590	1.0607	1.0015,1.1235	0.0475				
	Simple mode		0.0654	1.0676	0.9524,1.1968	0.2646				
Triglyceride levels in large HDLs in large HDL	IVW	56	0.0084	1.0084	0.9691,1.0493	0.6784	0.0022	0.3072	95.7139	0.0004
	MR Egger		−0.0117	0.9883	0.9352,1.0444	0.6779				
	Weighted median		−0.0145	0.9855	0.9438,1.0292	0.5113				
	Weighted mode		−0.0135	0.9865	0.9497,1.0247	0.4874				
	Simple mode		−0.0600	0.9417	0.8344,1.0627	0.3346				
Triglyceride levels in large LDL	IVW	69	0.0057	1.0057	0.9507,1.0639	0.8418	−0.0011	0.6818	189.2779	1.33E-13
	MR Egger		0.0199	1.0201	0.9340,1.1140	0.6592				
	Weighted median		−0.0159	0.9841	0.9358,1.0348	0.5327				
	Weighted mode		−0.0149	0.9852	0.9421,1.0301	0.5152				
	Simple mode		0.0248	1.0251	0.9140,1.1498	0.6722				
Triglyceride levels in large VLDL	IVW	65	0.0757	1.0787	1.0223,1.1382	0.0056	0.0002	0.9386	130.7291	1.19E-06
	MR Egger		0.0731	1.0758	0.9871,1.1725	0.1006				
	Weighted median		0.0825	1.0861	1.0224,1.1537	0.0073				
	Weighted mode		0.0614	1.0633	1.0025,1.1278	0.0449				
	Simple mode		0.0900	1.0942	0.9791,1.2227	0.1171				
Triglyceride levels in medium HDL	IVW	57	0.0440	1.0450	0.9952,1.0973	0.0766	0.0031	0.283	120.0518	9.58E-07
	MR Egger		0.0109	1.011	0.9358,1.0921	0.7820				
	Weighted median		0.0110	1.011	0.9549,1.0705	0.7054				
	Weighted mode		0.0122	1.0122	0.9599,1.0675	0.6539				
	Simple mode		0.0122	1.0122	0.9064,1.1305	0.8292				
Triglyceride levels in medium LDL	IVW	67	0.0414	1.0423	0.9835,1.1046	0.1613	−0.0007	0.7932	181.2587	6.46E-13
	MR Egger		0.0520	1.0534	0.9550,1.1618	0.3018				
	Weighted median		0.0086	1.0086	0.9536,1.0668	0.7624				
	Weighted mode		−0.0020	0.9979	0.9395,1.0600	0.9472				
	Simple mode		0.0406	1.0414	0.9252,1.1723	0.5031				
Triglyceride levels in medium VLDL	IVW	59	0.0655	1.0677	1.0096,1.1291	0.0215	−0.0003	0.9064	127.8589	2.34E-07
	MR Egger		0.0696	1.0721	0.9807,1.1721	0.1308				
	Weighted median		0.0411	1.0419	0.9807,1.1069	0.1830				
	Weighted mode		0.0469	1.0480	0.9875,1.1123	0.1272				
	Simple mode		0.0363	1.0370	0.9199,1.1690	0.5542				
Triglyceride levels in small HDL	IVW	70	0.0789	1.0821	1.0306,1.1361	0.0014	0.0013	0.5958	145.1006	1.62E-07
	MR Egger		0.0614	1.0633	0.9807,1.1529	0.1413				
	Weighted median		0.0642	1.0663	1.0048,1.1316	0.0340				
	Weighted mode		0.0559	1.0575	0.9990,1.1194	0.0579				
	Simple mode		0.0871	1.0910	0.9688,1.2287	0.1550				
Triglyceride levels in small LDL	IVW	68	0.0648	1.0670	1.0094,1.1278	0.0219	0.0022	0.4324	167.6241	8.52E-11
	MR Egger		0.0359	1.0366	0.9467,1.1350	0.4395				
	Weighted median		0.0364	1.0371	0.9789,1.0988	0.2152				
	Weighted mode		0.0342	1.0347	0.9817,1.0906	0.2070				
	Simple mode		0.0284	1.0289	0.9257,1.1435	0.5987				
Triglyceride levels in small VLDL	IVW	79	0.0734	1.0762	1.0246,1.1305	0.0034	0.000037	0.987	167.6065	1.12E-08
	MR Egger		0.0729	1.0757	0.9952,1.1626	0.0695				
	Weighted median		0.0555	1.0570	1.0002,1.1171	0.0491				
	Weighted mode		0.0431	1.0440	0.9865,1.1050	0.1398				
	Simple mode		0.0645	1.0667	0.9561,1.1900	0.2507				
Triglyceride levels in very small VLDL	IVW	76	0.0394	1.0402	0.9891,1.0940	0.1246	0.0002	0.9173	191.6806	2.20E-12
	MR Egger		0.0362	1.0369	0.9584,1.1219	0.3694				
	Weighted median		0.0048	1.0048	0.9564,1.0556	0.8482				
	Weighted mode		0.0129	1.0130	0.9678,1.0602	0.5804				
	Simple mode		0.0319	1.0324	0.9335,1.1417	0.5360				

### 3.5 The influence of the ratios of cholesterol and triglyceride within different lipoprotein particle sizes on IBS

Genetically determined cholesterol/free-cholesterol ratios in large HDL, large LDL, large VLDL, medium HDL, medium VLDL, small VLDL and very small VLDL were inversely associated with IBS, respectively (Table S7). Conversely, triglyceride ratios across the same particle spectrum consistently increased risk (OR 1.03–1.09, Table S9). These ratio analyses reinforce the divergent causal roles of cholesterol (protective) and triglycerides (detrimental) within overlapping lipoprotein pools (Tables S7, S8, S9).

## 4 Discussion

The present two-sample MR study clarifies the causal contributions of circulating lipids to IBS. Genetically elevated triglycerides—whether measured as total circulating levels or as cargo within any lipoprotein fraction—uniformly increased IBS risk. Conversely, cholesterol exhibited a diametrically opposed, particle-size-dependent relationship: harmful when carried by chylomicrons and extremely large VLDL, but protective when transported in very large HDL or very small VLDL particles. These divergent signals underscore the importance of lipoprotein heterogeneity beyond crude lipid class labels.

Lipoproteins are lipid–protein complexes whose buoyant density reflects their relative cargo of neutral lipids and apolipoproteins [14]. Chylomicrons deliver dietary triglycerides to peripheral tissues, VLDL supply endogenous triglycerides and, after lipolysis, generate remnant particles that are atherogenic precursors of LDL. Conversely, HDL mediates reverse cholesterol transport and protects against atherosclerosis [15,16]. Mendelian randomization has previously linked circulating triglycerides—rather than LDL- or HDL-cholesterol—to higher IBS risk [10,17]. By resolving lipoproteins into 13 size fractions we now show that the direction of the cholesterol–IBS relationship is particle-size-dependent. Genetically elevated cholesterol or free-cholesterol within chylomicrons or extremely large VLDL increased IBS risk, whereas equivalent increments in very-large HDL or very-small VLDL cholesterol were protective. These diametrically opposed effects indicate that the biological activity of cholesterol is dictated by its lipoprotein context, not by its absolute plasma concentration.

Accordingly, the same size-dependent biology that governs arterial inflammation appears to operate in the gut. Just as small, long-lived LDL particles penetrate the endothelium and promote atherosclerosis [18,19], they can translocate across a leaky intestinal epithelium, trigger mucosal macrophage activation and amplify visceral hypersensitive inflammatory responses as their oxidised derivatives [20], as the key features of IBS. Conversely, the rapid clearance of cholesterol-enriched, smaller VLDL or large HDL limits remnant accumulation within the lamina propria and delivers cholesterol to enterocytes for anti-inflammatory signalling [21]. Thus, lipoprotein size not only determines vascular fate, but also shapes the intestinal immune milieu, providing a mechanistic bridge between lipid metabolism and IBS pathophysiology.

Genetically elevated triglycerides within every VLDL sub-fraction uniformly increased IBS risk in our MR analysis, implying that the triglyceride moiety itself—rather than particle diameter—drives enteric pathology. The underlying mechanisms of this association could be multifactorial, potentially involving the induction of a low-grade inflammatory state in the gut by triglyceride-rich lipoprotein particles [22], alterations in the composition and functionality of the gut microbiota due to elevated triglyceride levels [23], or impacts on bile acid synthesis and other metabolic processes within the gut that could precipitate IBS [24].

In our MR analysis, both absolute cholesterol levels and the cholesterol-to-lipid ratio showed a protective effect against IBS leading us to propose that cholesterol—when carried by specific lipoproteins—may guard against the development of the disorder. We observed a size-dependent shift: genetically predicted cholesterol in very large VLDL was associated with increased risk, whereas the same cholesterol in very small VLDL was associated with reduced risk, This reversal can be attributed to two complementary mechanisms: (i) the adverse signal in large VLDL is driven primarily by their triglyceride-dominant composition, and (ii) cholesterol housed in smaller, cholesterol-enriched particlesis rapidly cleared and efficiently recycled into anti-inflammatory pathways rather than accumulating in the colonic mucosa [25]. Consequently, cholesterol plays a beneficial role only when compartmentalized within lipoproteins that direct it to regulatory, rather than pro-inflammatory, environments.

Beyond its canonical structural role, cholesterol is an obligate precursor for bile-acid, steroid-hormone and vitamin-D synthesis, processes indispensable for fat digestion, mineral metabolism and skeletal integrity [26,27]. Emerging evidence reveals broad anti-inflammatory and immunoregulatory properties that are equally relevant to mucosal homeostasis. Research has also explored the anti-inflammatory roles of cholesterol and its metabolic pathways: ABCA1/ABCG1-mediated efflux prevents NLRP3 inflammasome assembly and macrophage necrosis [28], whereas 25-hydroxycholesterol skews macrophages toward an anti-inflammatory phenotype and restrains NF-κB-dependent cytokine release [29]. Oxysterol-activated LXRs transcriptionally suppress TNF-α, IL-1β, IL-6, iNOS, COX-1, MMP-9, CCL2 and CCL7, extinguishing multiple inflammatory cascades [30,31]. Within the gut, apoA-I-containing HDL particles secreted by enterocytes sequester LPS and other bacterial products, thereby preserving gut-liver axis integrity [32]. Collectively, these pathways position cholesterol homeostasis as a tractable therapeutic node; enhancing cholesterol efflux already restores anti-tumour immunity in pre-clinical haematological models [33], underscoring the feasibility of harnessing cholesterol's immunoregulatory arm for mucosal diseases such as IBS.

### 4.1 Strengths & limitations

Although Cochran's Q revealed significant heterogeneity among the instrumental variables, we regard our conclusions as robust for three reasons. First, direction and magnitude of effect were consistent across all five MR estimators and across every lipoprotein fraction analysed. Second, the size-dependent switch in the cholesterol–IBS relationship (harmful in large VLDL/chylomicrons, protective in small VLDL/HDL) was replicated in an independent exposure dataset. Finally, leave-one-out and MR-Egger intercept analyses indicate that pleiotropy does not bias the primary IVW estimates, supporting the reliability of the observed associations.

## 5 Conclusion

In summary, our two-sample MR study provides genetic evidence that triglycerides uniformly increase IBS risk regardless of lipoprotein context, whereas cholesterol exerts a particle-size-dependent effect. These divergent causal pathways suggest that precision interventions that lower triglycerides while optimising cholesterol within anti-inflammatory particles may represent a rational, mechanism-based approach to prevent or ameliorate IBS.

## CRediT authorship contribution statement

**Fang Peng:** Methodology, Formal analysis, Data curation. **Kemin Xu:** Software, Investigation, Formal analysis. **Qinwen Ba:** Methodology. **Hao Bi:** Writing – review & editing, Data curation. **Yanjun Lu:** Writing – original draft, Supervision, Project administration.

## Ethics approval and consent to participate

Not applicable.

## Consent for publication

Not applicable.

## Availability of data and materials

Data are contained within the article and Supplementary Materials.

## Funding

This research received no external funding.

## Competing interests

The authors declare no conflicts of interest.

## Study concept and design: YJ, L. Data acquisition and analysis: FP. Interpretation of data: KM, X., QW, B. Abbreviations

IBS: Irritable bowel syndrome
GWAS: Genome-wide association studies
MR: Mendelian randomization
SNPs: Single nucleotide polymorphisms
VLDL: Very low-density lipoprotein
HDL: High-density lipoprotein
LDL: Low-density lipoprotein
IVW: Inverse variance weighted
OR: Odds ratios
CI: Confidence intervals
